# A novel enhanced stability detection algorithm for ablation catheters: purpose and application in high-power short-duration ablation

**DOI:** 10.3389/fcvm.2025.1556367

**Published:** 2025-07-04

**Authors:** Chiara Valeriano, Benjamin Berte, Ofer Klemm, Alona Sigal, Eid Zaknoun, Dimitri Buytaert, Tom De Potter

**Affiliations:** ^1^ Cardiovascular Center Aalst, Arrhythmia Unit, OLV Hospital, Aalst, Belgium; ^2^ Department of Advanced Biomedical Sciences, University of Naples Federico II, Naples, Italy; ^3^ Heart Center, Hirslanden St Anna, Lucerne, Switzerland; ^4^ R and D Department, Biosense Webster, Inc, Tirat HaCarmel, Israel

**Keywords:** atrial fibrallation, pulmonary vein isolation (PVI), stability+, high power short duration, radiofreqency ablation

## Abstract

**Background:**

Assessing catheter stability during ablation procedures is crucial. The current stability algorithm relies on end-expiration reference frame (Gated), requiring a full respiratory cycle before lesion tagging. This poses challenges with high-power, short-duration (HPSD) radiofrequency ablation workflows. To overcome these limitations, a novel algorithm, called Stability+, has been developed. It provides real-time tracking and analysis of catheter motion throughout the entire respiratory cycle.

**Objective:**

The aim of our study was to assess the performance of the new Stability + algorithm in HPSD ablations and to compare it with the current algorithm.

**Methods:**

Data from a series of consecutive left atrial ablations employing the new Stability + algorithm were prospectively collected. A retrospective analysis was conducted to compare the two algorithms.

**Results:**

A total of 1,056 applications were delivered, 123 (11.6%) using QMODE + (90 W, 3–4 s), and 933 (88.4%) using QMODE (50 W, ablation index guided 350/500). The number of unstable applications, outside the end-expiration phase, was detected with the Stability + for 9 positions (7.3%) using the QMODE+. Average time-to-tag appearance was 2.5 ± 1 s with the Stability + vs. 9 ± 1.1 s with the Gated algorithm. During QMODE ablation sessions, the Stability + algorithm prevented overshooting in 84% of the ablation positions. No steam pop or perforation occurred.

**Conclusion:**

The novel Stability + algorithm enhances lesion tracking for HPSD workflows like QMODE+/QMODE and holds the potential to improve stability detection across all radiofrequency ablation modes, marking a significant advancement in the field.

## Introduction

Catheter ablation for the treatment of atrial fibrillation (AF) has evolved tremendously over the past three decades, with pulmonary vein isolation (PVI) remaining the cornerstone of contemporary interventional treatment (1). Today, PVI is widely performed using 3D mapping systems in most centers, enabling non-fluoroscopic catheter manipulation, and providing valuable information about left atrial anatomy and its electrical characteristics (2). The VisiTag module, developed by Biosense Webster, Inc., is an automated ablation lesion tagging software integrated into the CARTO 3 system. It provides continuous storage, tracking, and quantification of catheter positions, along with the collection of ablation parameters during radiofrequency (RF) applications, with consistency in various clinical workflows across multiple centers (3).

The current algorithm (Gated) detects respiratory motion using an end-expiration reference frame for ablation tags. However, it does not track catheter motion outside this phase, which may overestimate catheter stability. Also, because it only analyzes end-expiration, a full respiratory cycle is needed before tagging and quantifying lesions. These limitations become particularly evident with recent ablation innovations that involve high-power short-duration (HPSD) settings, as the application often concludes before a complete respiratory cycle is completed. This has prompted the development of an enhanced algorithm (Stability+) with the primary objective of tracking and analyzing catheter motion throughout the entire respiratory cycle, delivering rapid feedback to the user, and achieving more precise instability detection. The aim of our study was to assess the performance of the Stability + algorithm in HPSD ablations and to compare it with the current algorithm.

## Methods

We conducted an initial feasibility study focusing on the technical behavior and procedural performance of the Stability + algorithm during left atrial HPSD ablations. We prospectively collected data from a series of 20 consecutive PVI conducted at a single center, employing the new Stability + algorithm. The following data points were analyzed for every RF application:

- **Ablation stability:** was calculated using the following parameters: catheter velocity <2.5 cm/sec and displacement distance <3 mm.
- **Time-to-tag appearance:** time to first ablation index (AI) indication. The AI target during QMODE ablations was 500 on the anterior wall and 350 on the posterior wall.

To facilitate a comparison between the two algorithms, we conducted a retrospective analysis and assessed the following metrics:

- The count of “unstable” applications occurring outside of the end-expiration phase.
- The average time-to-tag appearance.
- Ablation overshoot, denoting the number of ablation sessions in which the time taken to achieve the target AI of 350 exceeded the average time-to-tag appearance.

## Results

A total of 1,056 applications were delivered, QMODE + (90 W, 3–4 s) was used in 2 cases (123 applications, 11.6%) and QMODE (50 W, AI guided 350/500) in the remaining 18 cases (933 applications, 88.4%). All the procedure were performed in general anesthesia.

**Figure 1:**
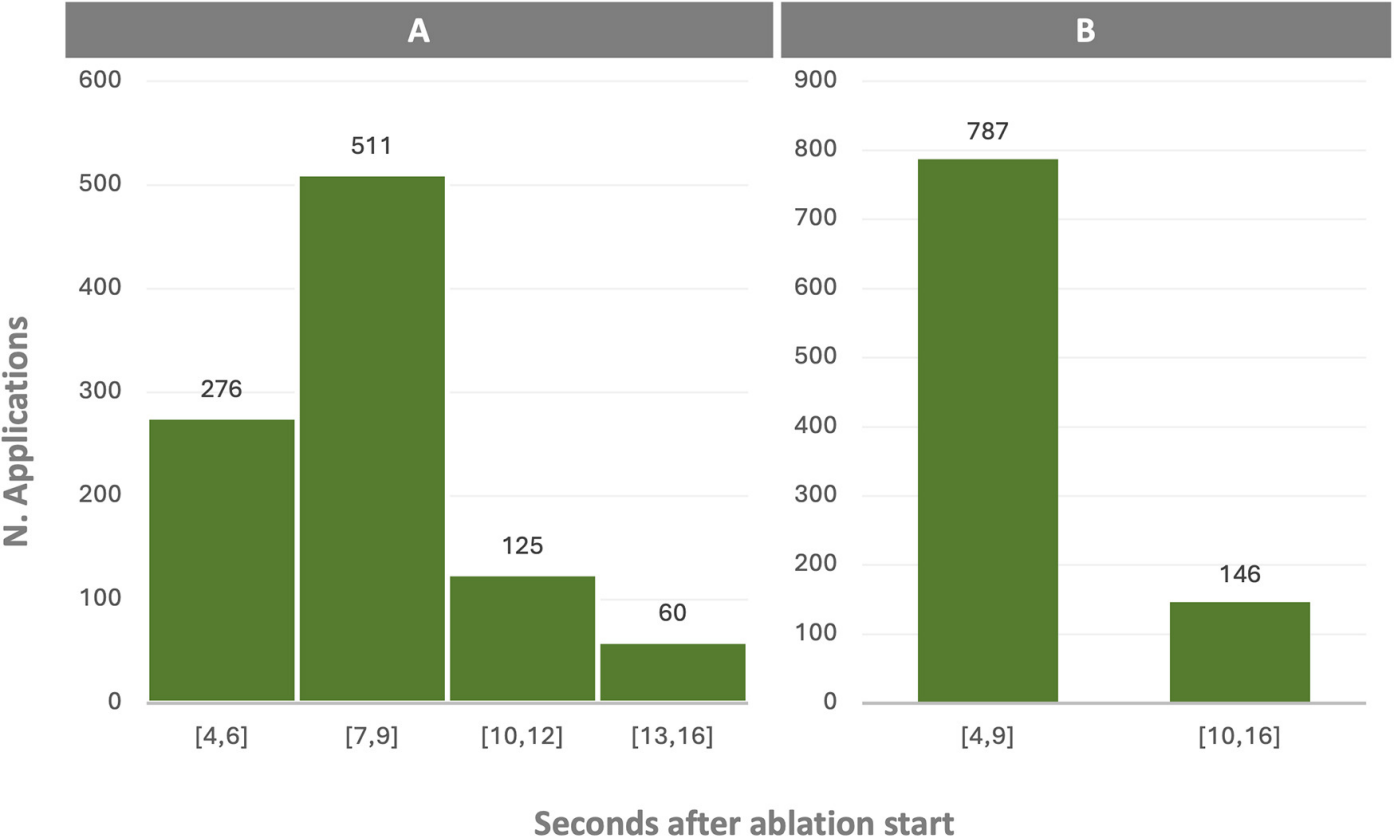
Time to achieve 350 ablation index target with stability + algorithm in QMODE: This bar chart illustrates the time it took to achieve a 350 ablation index (AI) target during ablation sessions conducted using the Stability + algorithm in QMODE. **(A)** Sessions are categorized by the time intervals needed to reach the 350 AI target. **(B)** Out of 933 sessions analysed, AI exceeded 350 within 9 s in 84% of the sessions. This implies that the desired AI level was reached more rapidly than the average time taken by the Gated algorithm to display a VisiTag with a standard respiration rate in 84% of the ablations, indicating overshoot.

- The number of “unstable” applications using the Stability + was identified for 19 positions (2.3%) using the QMODE and for 9 positions (7.3%) using the QMODE+.
- Average time-to-tag appearance was 2.5 ± 1 s with the Stability + vs. 9 ± 1.1 s with the Gated algorithm (*P* < 0.001), with a respiratory rate of 10–12 breaths per minute.
- Out of a total of 933 QMODE ablation sessions, AI reached the 350 threshold in less than 9 s, that is the average time-to-tag appearance with Gated algorithm, in 787 cases. This indicates that overshooting would have occurred before the AI indication display in 84% of the ablation positions with Gated algorithm (Figure 1).
- Pre-ablation stability, referring to catheter stability in the 2 s of pre-ablation irrigation, before the RF application effectively starts, was indicated in 961/1,056 (91%) of the ablation positions using the Stability + algorithm, QMODE 90% (840/933 applications) and QMODE + 98% (120/123 applications).

There were no occurrences of steam pop or perforation. No adverse events were recorded during the procedure, and there were no documented procedure-related complications during the hospital stay.

## Discussion

The data presented reflect our initial experience with a recently introduced stability detection system for PVI, demonstrating its feasibility and highlighting its primary advantages over current available technology, particularly in the context of HPSD ablation. The Stability + algorithm reduced the time-to-tag appearance to 2.5 s, thus drastically mitigating the risk of overshooting on the posterior wall when using QMODE, compared to the gated algorithm. It detected instability outside the end-expiration phase for 7.3% applications using QMODE+ and provided stability indications before the ablations begins, which allows the operator to optimize contact and positioning prior to RF energy delivery. This capability is especially valuable in workflows with RF applications lasting only 4 s.

**Figure 2:**
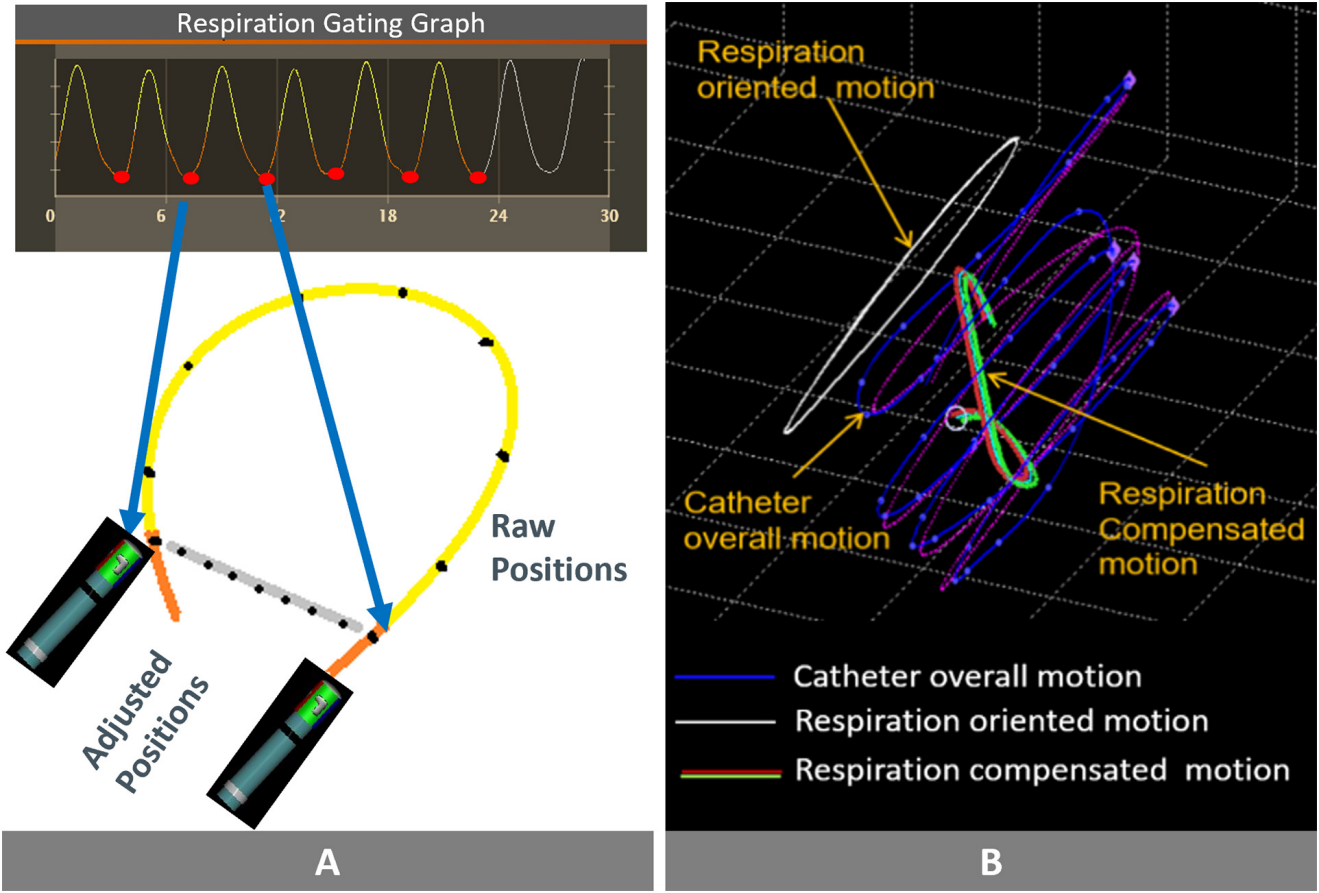
Comparing the two algorithms and their key features: **(A)** Respiratory Gated Algorithm: the red dots represent the positions of the catheter tip. The orange line connects two consecutive end-expiration phases, and stability is determined by measuring the difference in catheter tip positions between these points. The system does not take into account catheter tip location during this time (yellow tracing line). **(B)** Respiration Compensated Algorithm (Stability+): Overall catheter tip movement depicted by the purple line. The white line traces the impact of respiration on this overall movement. Subtracting the white line from the purple line yields the compensated catheter tip movement, indicated by the green line. Stability is based on the absolute value of the compensated catheter tip movement. Images are courtesy of © Biosense Webster, Inc. All rights reserved.

The Stability + algorithm continuously assesses respiratory-induced motion and adjusts the catheter's actual positions, providing real-time stability feedback. Unlike CARTO respiration training, which identifies end-expiration phases only during an initial calibration, Stability + maintains continuous correlation with respiratory motion throughout the entire procedure. This allows for respiratory compensation even in non-ventilated patients, or during apnea episodes. The key differences between the two algorithms are summarized in Table 1 and illustrated in Figure 2.

**Table 1:** Key differences: gated vs. stability + algorithm.

	Gated	Stability+
**Respiration training**	Manual	Automatic
**Maximum distance**	Based on the standard deviation distance between two consecutive end expiration positions	A continuous measurement of the absolute distance between the catheter's location and the center of mass of the compensated catheter position
**Temporal responsiveness**	Gated calculation	Continuous calculation
**Respiratory rate dependency**	Dependent, with potential delays in the appearance of Tags and corresponding ablation data	Independent, Tags and ablation data appearing within 2 s when the catheter is stable at the beginning of the ablation
**First site indication**	No indication if the minimum time threshold is not met	Immediate indication of the first site in an ablation session displayed as a white Tag
**Instability indication**	No	Yes, a ring (halo) appears around a site if it is considered unstable
**Short duration fit**	No	Yes

**Figure 3:**
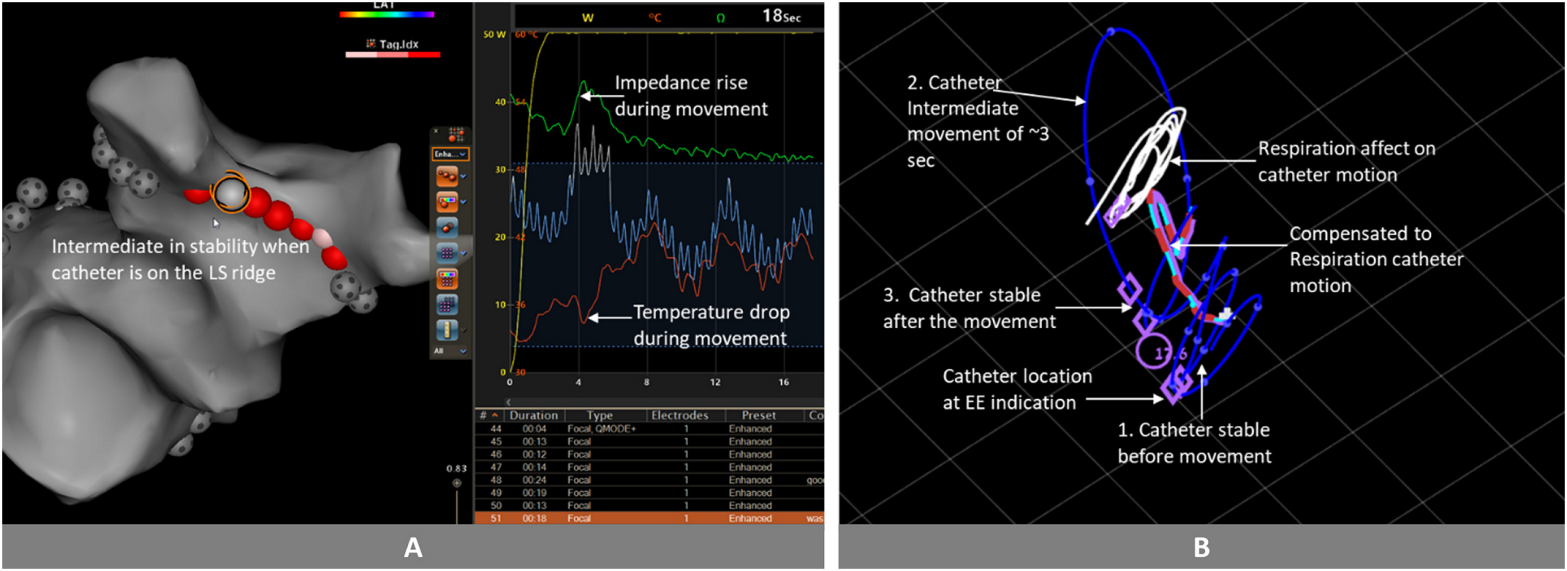
Catheter movement analysis and stability evaluation: **(A)** after few seconds of ablation, we observe a rise in impedance and a drop in temperature, indicating catheter movement. Approximately 3 s after this initial movement, the impedance continues to decrease, while the temperature continues to rise. **(B)** In the clinical analysis conducted in MATLAB, the catheter exhibited the following behavior: initially stable (1), subsequently moved for a duration of 3 s (2), returned close to its original position (3). Because the movement duration was brief, and the catheter returned sufficiently close to its initial position after the movement, accounting for thermal latency, the stability algorithm would classify the entire session as stable. Images are courtesy of © Biosense Webster, Inc. All rights reserved. Blue Line: Catheter tip trajectory; Blue Dots on Blue Line: 1 s interval markers; White Line: Respiration-induced catheter motion; Red-Light Blue Dashed Line: Compensated catheter motion; Purple Circle: Tag position at 17.6 s into the ablation; Purple Rhomboid: Catheter position at the end of the expiration phase.

Detecting catheter instability is the main goal when designing stability tracking algorithms. Striking the right balance between identifying clinically relevant instability and mere geometric variations is crucial. Excessive and erratic catheter movement, even if it returns to the same position by the end of expiration, can lead to suboptimal lesion formation. Conversely, brief deviations from the expected respiratory path, technically unstable on a millisecond scale, may not affect lesion efficacy due to thermal latency, a phenomenon in which tissue temperature continues to rise for approximately 3 s after RF termination (4). Stability + incorporates this consideration into its logic, preventing the misclassification of brief, non-impactful movements as instability (Figure 3).

The thermal latency phenomenon may be responsible for extramural lesions. One strategy to mitigate this risk is to target an AI of 350 on the posterior wall. This approach is supported by evidence indicating a lower incidence of thermal esophageal injury when the AI target value is limited to 350 compared to 380 (13.1% vs. 26.0%) (5). Our observations consistently linked the use of this 350 AI threshold with posterior wall overshooting when employing the Gated algorithm. The introduction of Stability + offers a promising solution for mitigating posterior wall overshooting—not only during PVI but also in the context of posterior wall isolation (PWI). Recent data have demonstrated higher success rates for PWI using vHPSD ablation (QMODE+) compared to standard power ablation (98% vs. 75%) (6). Our findings are reassuring for operators who routinely use the QMODE workflow and are already familiar with this setting. Further studies are needed to evaluate the performance of Stability + in posterior wall ablation across different power strategies (QMODE/QMODE+).

## Limitations

This is a single-center report with a small sample size, which only aims to describe and report potential advantages of a new stability algorithm specifically designed for HPSD workflows. As an initial feasibility and technical performance study, it was not powered or intended to assess long-term clinical outcomes such as arrhythmia recurrence or lesion durability. Moreover, the lack of a randomized design represents an inherent limitation and may introduce selection bias. Future investigations should evaluate the use of this new algorithm on a larger scale, including broader assessments of workflow efficiency, lesion quality, and its applicability across different anatomical regions.

## Conclusion

The novel Stability + algorithm enhances lesion tracking for HPSD workflows like QMODE+/QMODE and holds the potential to improve stability detection across all RF ablation modes, marking a significant advancement in the field. These findings provide a foundational basis for future clinical validation studies aimed to assess its influence on ablation outcomes.

## Data Availability

The raw data supporting the conclusions of this article will be made available by the authors, without undue reservation.
